# 10-Year Renal Function Trajectories in Community-Dwelling Older Adults: Exploring the Risk Factors for Different Patterns

**DOI:** 10.3390/jcm7100373

**Published:** 2018-10-20

**Authors:** Chia-Ter Chao, Yung-Ming Chen, Fu-Hui Ho, Kun-Pei Lin, Jen-Hau Chen, Chung-Jen Yen, COGENT Study Group (COhort of GEriatric Nephrology in NTUH)

**Affiliations:** 1 Department of Medicine, National Taiwan University Hospital BeiHu Branch, College of Medicine, National Taiwan University, Taipei 10617, Taiwan; b88401084@gmail.com; 2 Geriatric and Community Medicine Research Center, National Taiwan University Hospital BeiHu Branch, Taipei 10617, Taiwan; 3 Department of Internal Medicine; National Taiwan University, Taipei 10617, Taiwan; yungmingchen@ntu.edu.tw; 4 Department of Geriatrics and Gerontology, National Taiwan University Hospital, College of Medicine, National Taiwan University, Taipei 10617, Taiwan; diana@ntuh.gov.tw (F.-H.H.); dtmed512@gmail.com (K.-P.L.); jhhchen@ntu.edu.tw (J.-H.C.); 5 COohort of GEriatric Nephrology in National Taiwan University Hospital, Taipei 10617, Taiwan

**Keywords:** chronic kidney disease, estimated glomerular filtration rate, geriatrics, hematuria, proteinuria, sarcopenia

## Abstract

Longitudinal changes of renal function help inform patients’ clinical courses and improve risk stratification. Rare studies address risk factors predicting changes in estimated glomerular filtration rate (eGFR) over time in older adults, particularly of Chinese ethnicity. We identified prospectively enrolled community-dwelling older adults (≥65 years) receiving annual health examinations between 2005 and 2015 with serum creatinine available continuously in a single institute, and used linear regression to derive individual’s annual eGFR changes, followed by multivariate logistic regression analyses to identify features associated with different eGFR change patterns. Among 500 elderly (71.3 ± 4.2 years), their mean annual eGFR changes were 0.84 ± 1.67 mL/min/1.73 m^2^/year, with 136 (27.2%) and 238 (47.6%) classified as having downward (annual eGFR change <0 mL/min/1.73 m^2^/year) and upward eGFR (≥1 mL/min/1.73 m^2^/year) trajectories, respectively. Multivariate logistic regression showed that higher age (odds ratio (OR) 1.08), worse renal function (OR 13.2), and more severe proteinuria (OR 9.86) or hematuria (OR 3.39) were predictive of a declining eGFR while greater waist circumference (OR 1.06) and higher leukocyte counts (OR 1.21) were predictive of an uprising 10-year eGFR. These findings elucidate important features associated with geriatric renal function variations, which are expected to improve their renal care.

## 1. Introduction

Chronic kidney disease (CKD) has emerged as an important non-communicable disease worldwide, increasing the risk of adverse health outcomes including cardiovascular events and overall mortality [1]. However, it has been raised that the existing CKD classification schemes over-estimates the prevalence of CKD in older adults, and that part of those with advanced age demonstrate similar survival compared to younger ones [2,3]. Ageing attenuates the association between CKD, acute kidney injury, and the risk of impaired survival, with the magnitude of attenuation being augmented with higher age [4,5,6]. Moreover, factors that assist in predicting poor renal outcomes in the elderly have been controversial with poor consistency between studies. These issues pose a significant challenge to geriatricians and clinicians that provide care for the elderly.

Longitudinal changes of renal function, or estimated glomerular filtration rate (eGFR) trajectories, helps inform the clinical courses of patients with CKD, and the observation of eGFR trajectories offers important insights into the risk of developing end-stage renal disease (ESRD) in the future [7,8]. Reports from the CKD Prognosis Consortium disclosed that information gleaned from eGFR trajectories could help predict the risk of adverse renal outcomes, independent of the baseline eGFR [9]. Work done in diabetic patients even suggested that eGFR trajectories could displace baseline eGFR in prognostic modeling [10], lending support to the importance of tracing longitudinal eGFR changes in various populations. However, there have been few studies addressing risk features associated with eGFR trajectories among relatively healthy older adults, especially those with limited predictors for renal outcome. Existing studies have not provided adequate evidence guiding our care of these older adults, and there is even a group of “CKD regressors” exhibiting eGFR increase after years of follow-up [11]. Studies are urgently needed to identify factors that predict changes in eGFR over time in relatively healthy older adults [12], as are risk factor profiles that likely differ between individuals of diverse ethnicities [13]. 

Prior reports regarding eGFR trajectory among patients of non-Asian origin mostly focus on the description of patterns of eGFR trajectories in patients with different illnesses, risk factors for eGFR decline over time, and correlations between eGFR trajectories and distinct outcomes [14,15,16,17,18]. For studies investigating eGFR trajectories in Asian population, most of them deal with the pattern of eGFR trajectories in patients with diabetes mellitus (DM) [19,20], advanced CKD [21], and pediatric patients [22], while none of the existing literature addresses the patterns of eGFR trajectories and their associated risk factors in Asian elderly. Moreover, a recent study revealed that regression models based on all available eGFR values collected longitudinally estimate eGFR trajectory more accurately than the conventional two-point method [23]. Given this background, we hypothesized that the patterns of eGFR trajectories might differ depending on participants’ ethnic origin, and that certain clinical and laboratory features might correlate with different eGFR trajectory patterns among older adults of Chinese ethnicity. The present study was undertaken, by using regression modeling, to assess features associated with different eGFR change patterns in a prospectively maintained cohort of older adults who had received annual health examination services over an extended period of time.

## 2. Methods

### 2.1. Study Procedure

The annual elderly health examination in Taiwan included a comprehensive documentation of participants’ demographic profiles (age, gender), self-reported comorbidities, family history, and substance use history (tobacco, smoking, and betel nuts). Participants then received anthropometric measurements, including body height, body weight, body mass index (BMI), and waist circumference, with vital parameters (blood pressure (BP), and heart rate (HR)) recorded. These elderlies then received fasting blood tests for hemogram, serum biochemistry profiles including metabolic panels (glucose, cholesterol, uric acid), nutrition (total protein, albumin, globulin), liver (aspartate transaminase and alanine transaminase), and renal function (blood urea nitrogen and serum creatinine) [24]. Urinalysis was done using dipstick strips. Throughout the study period, serum creatinine was examined through the modified Jaffe method, as described previously [25,26].

We retrospectively identified community-dwelling older adults, defined as those with age ≥65 years, who received annual health examinations between 2005 and 2015 in the National Taiwan University Hospital, through a computerized search in our electronic database. We documented the baseline clinical features and laboratory data at their first encounter (year 2005). In order to examine the trend of renal function changes during the study period, we enrolled only those with serum creatinine available at the start (year 2005) and the end (year 2015) of this study for analysis, and recorded all their serum creatinine data during the entire study period. We calculated eGFR based on the four-variable Modification of Diet in Renal Disease (MDRD) formula, which was the standard eGFR calculation formula used during this period.

### 2.2. Analyzing the Trend of eGFR Changes among Participants Divided into Different Groups

After obtaining serum creatinine and calculating eGFR, we plotted the eGFR data points of each participant during the study period onto a figure using Excel software (Microsoft Corporation, Redmond, USA), with *x*- and *y*-axis of year and eGFR in mL/min/1.73 m^2^, respectively. We then conducted a simple linear regression to derive the slope of annual eGFR change for each participant. A distribution curve was used to illustrate the data range. We classified participants into three groups, the reference group, the biochemical eGFR decrease group, and the biochemical eGFR increase group, based on the thresholds selected using the distribution curve.

Following patient classification, we compared the demographic profiles, comorbidities, substance use, anthropometric and physical parameters, and blood and urine test results between the three groups of patients. Multivariate logistic regression analyses, the biochemical eGFR increase or decrease group as the dependent variable, incorporating demographic, comorbidities, substance history, anthropometric and physical parameters, and blood and urine tests were conducted to identify risk factors associated with different patterns of trajectory changes. We examined the areas under receiver-operating-characteristic (AUROC) curves to determine model validity.

### 2.3. Statistical Analysis

Statistical analyses were performed using the SPSS 18th version (IBM Corporation, Chicago, IL, USA). Continuous and categorical variables were presented in mean ± standard deviation and numbers with percentages, respectively. The clinical features of the three groups (reference, increase, and decrease) were compared using the analysis of variance (ANOVA). A two-tail *p*-value lower than 0.05 was considered significant.

### 2.4. Ethical Approval

The institutional review board of National Taiwan University Hospital approved the current study (No. 201807001RINB), and its protocol adheres to the Declaration of Helsinki. Their identification information was removed before data retrieval and, thus, informed consent was waived by the institutional review board due to data anonymity.

## 3. Results

A total of 500 older adults (age ≥65 years) with available year 2005 and 2015 serum creatinine data were identified and entered into subsequent analysis. The mean age of the enrollees were 71.3 ± 4.2 years, with 52% male (Table 1). The most common comorbidity was hypertension (46%), followed by hyperlipidemia (31%) and cardiovascular disease (21%). Their mean eGFR at baseline was 72.7 ± 15 mL/min/1.73 m^2^, with totally 4518 serum creatinine obtained. Each participant received on average 9 ± 1.7 times of serum creatinine measurement during the study period.

**Figure 1:**
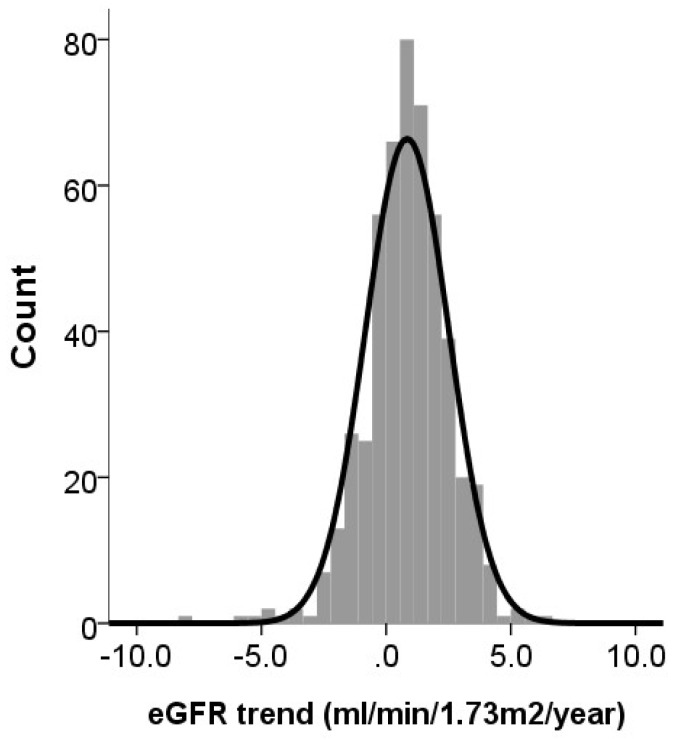
The distribution curve of annual eGFR changes among the entire cohort. eGFR, estimated glomerular filtration rate.

The mean and median annual eGFR changes among study participants were 0.84 ± 1.67 and 0.91 mL/min/1.73 m^2^/year, respectively, and the results conformed to normal distribution (Figure 1). 364 (72.8%) had positive eGFR changes during the 10-year period. Six (1.2%) and three (0.6%) of them had annual eGFR changes <−4 and <−5 mL/min/1.73 m^2^/year, respectively, while 12 (2.4%) and four (0.8%) had annual eGFR changes ≥4 and ≥5 mL/min/1.73 m^2^/year, respectively. Based on the mean/median levels and prior reports [27], we defined the reference group as those with an annual eGFR change between 0 and 1 mL/min/1.73 m^2^/year. Older adults with an annual eGFR change <0 or ≥1 mL/min/1.73 m^2^/year were assigned to the biochemical eGFR decrease and increase group, respectively. According to this classification, 126 (25.2%) served as the reference group, while 136 (27.2%) and 238 (47.6%) belonged to the decrease and the increase eGFR groups, respectively (Table 1). We found that participants of the decrease and the increase eGFR groups had significantly higher and lower age than the reference patients (both *p* < 0.01), and the decrease group were more likely to be male while the increase group were less likely (both *p* < 0.01) (Table 1). Participants of the decrease group had a significantly higher prevalence of DM (*p* < 0.01), higher systolic BP (*p* < 0.01), eGFR (*p* < 0.01), fasting glucose (*p* < 0.01), and uric acid (*p* = 0.01) compared to the reference and the increase group, while participants of the eGFR increase group had a higher serum globulin (*p* = 0.03) and glucose (*p* < 0.01) compared to the reference group (Table 1). We also showed that the decrease group exhibited higher levels of proteinuria (*p* = 0.02) and hematuria (*p* < 0.01) than those of the reference and the increase group. The eGFR trajectories did not differ between those with and without smoking (with vs. without, 0.78 ± 1.34 vs. 0.86 ± 1.68 mL/min/1.73 m^2^/year, *p* = 0.89) or those with and without alcohol consumption (with vs. without, 0.74 ± 1.48 vs. 0.91 ± 1.74 mL/min/1.73m^2^/year, *p* = 0.31) status.

Multivariate logistic regression analyses incorporating all variables in Table 1, with the decrease eGFR group as the dependent variable, was performed to examine risk factors for having biochemical eGFR decrease over the study period. We found that baseline features of advanced age (odds ratio (OR) 1.08), hyperglycemia (OR 1.04), worse renal function (OR 13.2), and more severe proteinuria (OR 9.86) or hematuria (OR 3.39) were associated with a higher risk of eGFR decrease over 10 years (Table 2); on the other hand, higher hemoglobin levels were independently associated with lower risk over 10 years (Table 2). The AUROC of this logistic regression model was 0.79 (95% CI 0.74–0.84). We also investigated associated factors for having biochemical eGFR increase during the study period, and showed that a baseline greater waist circumference (OR 1.06) and higher leukocyte counts (OR 1.21) were associated with a higher probability of biochemical eGFR increase over 10 years (Table 3). On the contrary, higher age (OR 0.92), being male (OR 0.5), and having a baseline higher BMI (OR 0.85) predicted a lower probability of biochemical eGFR increase after follow-up (Table 3). The AUROC of this logistic regression model was 0.69 (95% CI 0.62–0.74).

A sensitivity analysis was done to test different cutoffs for grouping eGFR trajectories. We re-divided the enrolled participants based on annual eGFR changes <25%, 25% to 75%, and ≥75%, and assigned them as the eGFR decrease group, reference group, and the eGFR increase group, respectively. The 25% and 75% cutoff values were −0.12 and 1.9 mL/min/1.73 m^2^/year, respectively. Multivariate regression analyses revealed that baseline higher age (OR 1.11), urine OB (OR 3.28), serum glucose (OR 1.03), and creatinine were similarly associated with a higher risk of biochemical eGFR decrease over 10 years, while baseline cardiovascular comorbidity emerged as a new risk factor (supplementary Table S1). Higher age (OR 0.91) was still associated with a lower probability of biochemical eGFR increase over time (supplementary Table S2).

## 4. Discussion

In the current study, we showed that eGFR could change slowly over an extended length of time in a group of community-dwelling older Chinese adults with little comorbidity. The average eGFR change was 0.84 mL/min/1.73 m^2^ per year, with 72.8% of participants having biochemical eGFR increase during follow-up. Divided into the reference, the eGFR increase, and the eGFR decrease groups, we found that higher age, poorer baseline renal function, hyperglycemia, urinalysis abnormalities, including proteinuria and hematuria, were important risk features associated with biochemical eGFR decrease, while lower age, BMI, higher waist circumference, and leukocyte counts were factors associated with biochemical eGFR increase. The identification of these risk features can be an integral step toward uncovering factors influencing renal function variation in the elderly, and can help us devise potential strategies to mitigate the detrimental effects brought by this fluctuation.

The annual eGFR changes in our elderly participants seem to differ from those reported by others in the literature. Salimi et al. reported that the mean rate of annual eGFR change was −0.3 ± 0.1 mL/min/1.73 m^2^/year among non-diabetic community-dwelling elderly from United States during a 9-year period [28], while patients of African American ethnicity exhibited an annual eGFR change of −1.27 mL/min/1.73 m^2^ over more than 8 years of follow-up [29]. Comparing our findings to theirs, it is evident that the annual eGFR changes among our participants are more likely to be positive and had higher absolute values than those reported in other studies, and the proportion of older adults with greater annual eGFR changes is similarly higher in our study compared to those reported in Caucasian patients. Part of these older adults may exhibit a trend of successful aging, leading to a stable renal function over an extended period. In addition, we further propose several reasons to account for this discrepancy. First, our enrolled cohort consisted of independently living older adults capable of receiving annual health examination consecutively for 10 years (alive at last), and presumably those exhibiting greater eGFR decrease over time might succumb to mortality or develop ESRD, preventing them from receiving further health examinations. In this sense, the possibility of a preferential selection of healthier older adults cannot be excluded. Second, a relatively high proportion of our older participants had on average biochemical eGFR increase over time, positively affecting the overall eGFR trajectories. Finally, the possibility exists that some of these older adults sustained inter-current illnesses during the extended study period, and acute illnesses are known to alter body volume kinetics and lead to falsely low creatinine levels with increased eGFR levels [30]. This may skew the eGFR change rates toward positivity, although we believe that the influence of these factors is also low since the disease severity should be low enough to permit participants’ return for subsequent health examination.

The approach used in this study to estimate longitudinal eGFR change also warrants attention. Some researchers indicated that longitudinal eGFR changes tended to be non-linear when patients being observed had poorer renal function or the observation period was prolonged, and sophisticated analytical methods could be used to overcome this approximation difficulty [31]. Prior reports attempted to use pattern recognition, percent annual decline, eGFR stage changes, or latent class modeling for classifying eGFR trajectories, with most of their utility demonstrated [16,32]. Most studies agree that more than two serum creatinine data points per individual are needed to better approximate the trend of eGFR changes over time [33]. In our study, we used linear regression modeling to estimate eGFR trajectories, an approach that has been adopted by many in the past with good outcome-correlation shown [7,8,9]. In addition, we found that only 14.6% of our participants had wide fluctuation of eGFR levels over the study period (defined as having any data points between 2005 and 2015 >10 mL/min/1.73 m^2^ increase or decrease from their start or end eGFR levels). Consequently, we believe that a linear regression approach can reasonably approximate the secular eGFR trend in the participants of this cohort.

An interesting finding of this study is that part of the older adults had biochemical eGFR increase during extended follow-up, although the magnitude of increase was mostly low (75% < 2 mL/min/1.73 m^2^/year). Renal function are expected to decline progressively with age, but this trend may stall or rarely be reversed. We believe that the most important and plausible reason to explain this phenomenon of secular biochemical eGFR increase is the influence of age-related sarcopenia. A population-based survey in Asian population has shown that being sarcopenic and sarcopenic obese based on dual energy X-ray absorptiometry (DXA) is independently associated with having a high eGFR levels [34]. Furthermore, studies identifying a paradoxical association between higher creatinine-based eGFR and increased mortality are frequently justified based on the participants’ low lean body mass and sarcopenic/cachetic status, with relationship nullified or reversed after adjusting for muscle indices [10,35]. In patients with CKD, there are also a group of regressors with positive eGFR slopes not readily explained by measurement variations [11]. In this study, we showed that lower BMI and higher waist circumference were associated with a higher probability of biochemical eGFR increase in these older adults (Table 3); indeed, lower BMI might be a surrogate for lower lean body mass while increasing waist circumference could indirectly suggest obesity [36]. The association between serum leukocyte counts and a higher probability of biochemical eGFR increase could also be explained by the presence of malnutrition-inflammation-cachexia syndrome in older adults, as shown by others [37]. The influence of age-related lean body mass loss, or sarcopenia, may be more prominent in older adults of Asian ethnicity compared to Caucasians, and this can potentially explain why the phenomenon of biochemical eGFR increase is rarely reported in studies done in older adults from the Western countries [13].

We showed that higher age, serum creatinine, proteinuria, hematuria, and hyperglycemia were associated with biochemical eGFR decrease among older adults (Table 2), and these risk features have been repetitively shown to predict a poorer overall and renal outcome among participants with or without CKD [24,38,39]. We enrich the existing literature by showing that factors negatively influencing renal health in the general population work similarly in the elderly with a greater magnitude, and this effect is independent of their baseline renal function. We previously showed that dipstick proteinuria was predictive of functional impairment among older adults [40], and in combination with results from this study, a trajectory of renal function decline may correlate with subsequent functional impairment independently. Nonetheless, this proposition needs to be confirmed in prospective studies in the future.

This study has its strength and limitations. The longitudinal nature of our study, the regularity of serologic examinations over time (mean 9 tests/10 years), and the relatively lower variation of eGFR during the study period lend support to the credibility of our findings. Our study possess advantages including the *a priori* focus on the associated factors of eGFR trajectory in elderly of Asian origin, which fills the research gap of the existing literature, and the extended follow-up period (10-year), which avoids the interference of eGFR variability and transient renal injury episodes. However, our results need to be interpreted cautiously in light of its background, including the context in which study participants were enrolled (annual health examinations), the eGFR formula being used to estimate renal function, and the participants’ ethnicity origin (all ethnic Chinese). We did not test for novel renal function markers such as neutrophil gelatinase-associated lipocalin (NGAL), rendering ascertainment of renal function change impossible. In addition, we chose the eGFR slope arbitrarily based on the slope distribution among our participants, which might not be applicable to other geriatric populations. However, similar cutoffs have been utilized before by others in categorizing eGFR trajectories, with credible results obtained. Studies based on more patient numbers and with different eGFR modeling techniques may be needed to confirm our findings.

## 5. Conclusions

In conclusion, using a cohort of community-dwelling older adults with consecutive renal function follow-up, we showed that their annual eGFR changes were 0.84 mL/min/1.73 m^2^/year over 10 years. Higher age, serum creatinine and glucose, having proteinuria or hematuria portended a higher risk of annual eGFR decline <0 mL/min/1.73 m^2^ among older adults, while those with baseline features of sarcopenia or sarcopenic obesity were more likely to exhibit the trend of biochemical eGFR increase during follow-up. The identification of these risk features is expected to improve the renal care of older adults and optimize treatment strategy directed toward those at risk of renal function variation in the future.

## Figures and Tables

**Table 1:** Comparison of clinical features between community-dwelling elderly with different trajectories of annual estimated glomerular filtration rate (eGFR) changes (in mL/min/1.73 m^2^).

Clinical Features	Total (*n* = 500)	Annual Change < 0 (*n* = 136)	Reference Group (*n* = 126)	Annual Change ≥ 1 (*n* = 238)	*p*-Value
Demographic profile					
Age (years)	71.3 ± 4.2	72.4 ± 4.6	71.7 ± 3.9	70.4 ± 4	<0.01
Gender (male%)	259 (52)	84 (62)	70 (56)	105 (44)	<0.01
Smoking (%)	31 (6)	17 (13)	1 (1)	13 (5)	0.02
Alcohol (%)	167 (33)	52 (38)	51 (40)	64 (27)	0.03
Anthropometric parameters					
Body height (cm)	160.5 ± 8.2	161.2 ± 7.9	160.7 ± 8.1	159.9 ± 8.4	0.31
Body weight (kg)	61.4 ± 9.7	62.5 ± 9.4	61.4 ± 9.3	60.7 ± 10.1	0.23
BMI (kg/m^2^)	23.8 ± 3.1	24 ± 3.1	23.7 ± 3.1	23.7 ± 3.1	0.56
Waist circumference (cm)	84.8 ± 9.7	86 ± 10.2	83.8 ± 10.1	84.6 ± 9	0.15
Other comorbidities by history					
Hypertension (%)	229 (46)	69 (51)	59 (47)	101 (42)	0.29
Diabetes mellitus (%)	41 (8)	21 (15)	6 (5)	14 (6)	<0.01
Cardiovascular disease (%)	107 (21)	38 (28)	25 (20)	44 (18)	0.09
Hyperlipidemia (%)	155 (31)	45 (33)	43 (34)	67 (28)	0.42
Gout/hyperuricemia (%)	53 (11)	19 (14)	17 (13)	17 (7)	0.06
Chronic kidney disease (%)	1 (0)	1 (1)	0 (0)	0 (0)	0.26
Chronic liver disease (%)	49 (10)	11 (8)	14 (11)	24 (10)	0.7
Chronic lung disease (%)	37 (7)	11 (8)	10 (8)	16 (7)	0.86
Dementia (%)	0 (0)	0 (0)	0 (0)	0 (0)	-
Physical parameters					
Systolic BP (mmHg)	133.2 ± 15.9	136.9 ± 18.6	132.2 ± 15.3	131.6 ± 14	<0.01
Diastolic BP (mmHg)	78 ± 10.7	79 ± 11.5	77.4 ± 10.3	77.7 ± 10.3	0.42
Heart rate (/minute)	70.3 ± 9.7	69.5 ± 9.5	69.6 ± 10.1	71.1 ± 9.6	0.2
Blood test results					
Hemoglobin (g/dL)	13.7 ± 1.2	13.7 ± 1.2	13.8 ± 1.2	13.7 ± 1.2	0.58
Platelet (×10^3^/µL)	207.8 ± 47.2	205.2 ± 49.5	202.4 ± 47.7	212.2 ± 45.4	0.13
Leuokocyte (×10^3^/µL)	5.6 ± 1.3	5.6 ± 1.2	5.4 ± 1.4	5.7 ± 1.3	0.11
Total protein (mg/dL)	7.3 ± 0.4	7.3 ± 0.4	7.3 ± 0.4	7.3 ± 0.4	0.34
Albumin (mg/dL)	4.5 ± 0.2	4.5 ± 0.2	4.5 ± 0.2	4.5 ± 0.2	0.24
Globulin (mg/dL)	2.8 ± 0.4	2.8 ± 0.3	2.8 ± 0.3	2.9 ± 0.4	0.03
Urea nitrogen (mg/dL)	16.5 ± 3.9	17.4 ± 4.2	16.4 ± 3.6	16 ± 3.7	<0.01
Creatinine (mg/dL)	0.98 ± 0.2	0.99 ± 0.3	1 ± 0.2	0.97 ± 0.2	0.43
eGFR (mL/min/1.73 m^2^)	72.7 ± 15	76.4 ± 20.1	71.2 ± 12.8	71.4 ± 12	<0.01
Fasting glucose (mg/dL)	99.7 ± 16.8	105.3 ± 23.1	96.4 ± 13.3	98.3 ± 13.1	<0.01
Total cholesterol (g/dL)	195 ± 32.8	190.4 ± 34.2	192.9 ± 33.4	198.7 ± 31.3	0.05
Triglyceride (mg/dL)	113.3 ± 58.6	119.8 ± 67.1	106.6 ± 51.6	113.2 ± 56.7	0.19
Uric acid (mg/dL)	6.1 ± 1.4	6.3 ± 1.4	6.2 ± 1.4	5.9 ± 1.4	0.01
Urinalysis results					
Occult blood (0–4+)	0.1 ± 0.36	0.19 ± 0.49	0.06 ± 0.29	0.07 ± 0.29	<0.01
Protein (0–4+)	0.05 ± 0.29	0.11 ± 0.43	0.03 ± 0.2	0.03 ± 0.22	0.02

BMI, body mass index; BP, blood pressure; CKD, chronic kidney disease; eGFR, estimated glomerular filtration rate; -: based on the four-variable Modification of Diet in Renal Disease (MDRD) formula.

**Table 2:** Multivariate logistic regression modeling with annual eGFR change < 0 mL/min/1.73 m^2^ as the dependent variable.

Variable	Odds Ratio	95% Confidence Interval	*p*-Value
Age (year)	1.08	1.01–1.16	0.03
Diastolic blood pressure (mmHg)	1.03	0.998–1.05	0.07
Urine protein (titer)	9.86	1.42–68.4	0.02
Urine OB (titer)	3.39	1.3–8.8	0.01
Hemoglobin (g/dL)	0.74	0.56–0.97	0.03
Glucose (mg/dL)	1.04	1.02–1.06	<0.01
Creatinine (mg/dL)	13.2	1.13–153.7	0.04

eGFR, estimated glomerular filtration rate; OB, occult blood.

**Table 3:** Multivariate logistic regression modeling with annual eGFR change ≥ 1 mL/min/1.73 m^2^ as the dependent variable.

Variable	Odds Ratio	95% Confidence Interval	*p*-Value
Age (year)	0.92	0.87–0.97	<0.01
Male gender	0.5	0.3–0.82	<0.01
BMI (kg/m^2^)	0.85	0.75–0.96	<0.01
Waist circumference (cm)	1.06	1.02–1.11	<0.01
Leukocyte (K/µL)	1.21	1.01–1.45	0.04

BMI, body mass index; eGFR, estimated glomerular filtration rate.

## Supplementary Materials

The following are available online at http://www.mdpi.com/2077-0383/7/10/373/s1. **Supplementary Table S1.** Sensitivity analysis using multivariate logistic regression modeling with biochemical eGFR decrease group as the dependent variable, incorporating different grouping criteria. **Supplementary Table S2.** Sensitivity analysis using multivariate logistic regression modeling with biochemical eGFR increase group as the dependent variable, incorporating different grouping criteria.
