# Supplementary material for: 10-Year Renal Function Trajectories in Community-Dwelling Older Adults: Exploring the Risk Factors for Different Patterns
Source: J Clin Med. 2018 Oct 20;7(10):373. doi: 10.3390/jcm7100373 (PMC6210637; doi:10.3390/jcm7100373)
Supplement: Supplementary file 1 [file jcm-07-00373-s001.pdf]

Supplementary Table S1. Sensitivity analysis using multivariate logistic regression modeling with biochemical eGFR decrease group as the dependent variable, incorporating different grouping criteria

Variable	Odds ratio	95% confidence interval	*P value*
Age (year)	1.11	1.05 – 1.18	*< 0.01*
Male gender	0.16	0.05 – 0.53	*< 0.01*
Cardiovascular disease	1.81	1.02 – 3.2	*0.02*
Urine OB (titer)	3.28	1.66 – 6.5	*0.01*
Glucose (mg/dL)	1.03	1.01 – 1.04	*< 0.01*
Creatinine (mg/dL)	1.2×10^4	142 – $1x\ 10^6$	*0.04*

eGFR, estimated glomerular filtration rate; OB, occult blood

Supplementary Table S2. Sensitivity analysis using multivariate logistic regression modeling with biochemical eGFR increase group as the dependent variable, incorporating different grouping criteria

Variable	Odds ratio	95% confidence interval	*P value*
Age (year)	0.91	0.86 – 0.97	*< 0.01*
Diabetes mellitus	0.31	0.09 – 1.08	*0.07*
Uric acid (mg/dL)	0.82	0.69 – 0.97	*0.02*

BMI, body mass index; eGFR, estimated glomerular filtration rate
